# Predictors of neurological outcomes after successful extracorporeal cardiopulmonary resuscitation

**DOI:** 10.1186/s12871-015-0002-3

**Published:** 2015-03-08

**Authors:** Jeong-Am Ryu, Yang Hyun Cho, Kiick Sung, Seung Hyuk Choi, Jeong Hoon Yang, Jin-Ho Choi, Dae-Sang Lee, Ji-Hyuk Yang

**Affiliations:** 1 Department of Critical Care Medicine, Samsung Medical Center, Sungkyunkwan University School of Medicine, Seoul, South Korea; 2 Department of Thoracic and Cardiovascular Surgery, Samsung Medical Center, Sungkyunkwan University School of Medicine, Seoul, South Korea; 3 Division of Cardiology, Department of Medicine, Samsung Medical Center, Sungkyunkwan University School of Medicine, Seoul, South Korea; 4 Department of Emergency Medicine, Samsung Medical Center, Sungkyunkwan University School of Medicine, Seoul, South Korea

**Keywords:** Extracorporeal membrane oxygenation, Extracorporeal life support, Cardiopulmonary resuscitation, Cardiac arrest

## Abstract

**Background:**

Extracorporeal cardiopulmonary resuscitation (ECPR) refers to use of extracorporeal membrane oxygenation (ECMO) in cardiopulmonary arrest. Although ECPR can increase survival rates after cardiac arrest, it can also result in poor post-resuscitation neurological status. Thus, we investigated predictors of good neurological outcomes after successful ECPR.

**Methods:**

A total of 227 patients underwent ECPR from May 2004 to June 2013 at Samsung Medical Center. Successful ECPR was defined as survival more than 24 hours after ECPR. Neurological outcomes were assessed at discharge using the Glasgow-Pittsburgh Cerebral Performance Categories scale (CPC). CPC 1 and 2 were classified as good and CPC 3 to 5 were classified as poor neurological outcomes. Excluded were 22 patients who did not survive more than 24 hours after ECPR and 90 patients who died from unknown causes or causes other than brain death or whose neurological status could not be assessed at discharge. Multiple logistic regression analysis was used to identify independent predictors of neurological outcomes.

**Results:**

Included were 115 patients with a mean age of 58 (range 45–66) years and 80 men (70%). Cardiopulmonary resuscitation (CPR) was performed at non-hospital sites for 19 (17%) patients and bystander CPR was performed in 9 of 19 cases (47%). Cardiac etiology was verified in 74 (64%) patients and therapeutic hypothermia was performed in 9 patients (8%); 68 (59%) had good neurological outcomes and 47 (41%) did not and 24 patients died from brain death. Neurological outcomes were affected by hemoglobin levels before ECMO (*P* = 0.02), serum lactic acid (*P* < 0.001) before ECMO insertion, and interval from cardiac arrest to ECMO (*P* = 0.04).

**Conclusions:**

Low hemoglobin or high serum lactic acid levels before ECMO, and prolonged interval from cardiac arrest to ECMO predicted poor neurological outcomes after successful ECPR. Early institution of ECMO and a low threshold for blood transfusion might improve neurological outcomes for patients who survive ECPR.

## Background

Extracorporeal membrane oxygenation (ECMO) is a useful intervention for refractory cardiogenic shock and respiratory failure [1,2]. Because ECMO implementation can rapidly normalize circulation in patients under cardiac arrest, it has been used to assist cardiopulmonary resuscitation (CPR) [3-6]. Using traditional chest compression is less effective than using ECMO with CPR (known as extracorporeal CPR or ECPR). ECPR can achieve more effective recovery of spontaneous circulation (ROSC) than conventional CPR. ECMO implementation takes at least several minutes when cardiopulmonary arrest is persistent, even with conventional CPR. Since the brain is the organ most vulnerable to hypoxia and inadequate perfusion, ECPR can result in severe neurologic deficits if ECMO is not performed promptly [7]. In addition to delay, several factors may lead to poor neurological outcomes after ECPR. Achieving good neurological outcomes and successful resuscitation are important, so we investigated predictors of favorable neurological outcomes rather than survival after ECPR.

## Methods

This retrospective study was performed in a cohort of patients who underwent ECPR during hospitalization in Samsung Medical Center (a 1961-bed, university-affiliated, tertiary referral hospital in Seoul, South Korea). The study was approved by the Institutional Review Board of Samsung Medical Center (IRB No. SMC 2014-03-174-001) according to the Declaration of Helsinki on reviewing and publishing information from patients’ records. Informed consent was waived because of the retrospective nature of the study.

### Patients

**Figure 1:**
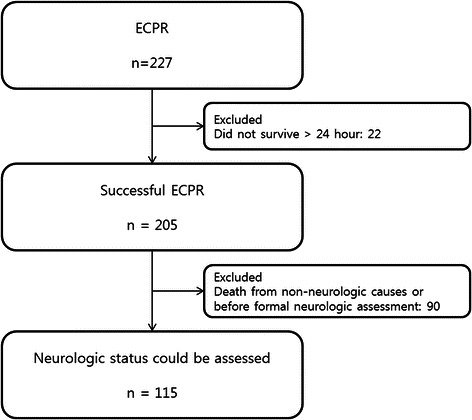
**Population inclusion and exclusion criteria.** ECPR, extracorporeal cardiopulmonary resuscitation.

From May 2004 to June 2013, 227 adult patients underwent ECPR at Samsung Medical Center. ECPR was defined as use of venoarterial ECMO intended to treat cardiac arrest. Successful ECPR was defined as survival longer than 24 hours after ECPR. Excluded were 22 patients who did not survive more than 24 hours after ECPR. Some patients who needed continuous sedation because of hemodynamic instability could not be awakened and spontaneous awakening trials were not possible. Some patients died from non-neurologic causes such as multiorgan failure, cardiac death, or uncontrolled infection. We excluded patients for whom we could not define neurological status because of continuous sedation or death from unknown causes or causes other than brain death. This resulted in 90 patients excluded and 115 patients included (Figure 1).

### Endpoints and definitions

Our primary endpoint was neurological outcome at hospital discharge, assessed with the Glasgow-Pittsburgh Cerebral Performance Categories (CPC) scale (1 to 5, as shown in Table 1) [8]. CPC 1 and 2 were classified as good neurological outcomes. CPC 3, 4, and 5 were considered poor neurological outcomes. We thoroughly reviewed medical records and patients were assigned to the CPC scale upon agreement by two authors (JAR and YHC). ECPR was defined ECMO performed during CPR. CPR duration was defined as time from chest compression onset to offset. ROSC was defined for all rhythms as restoration of spontaneous perfusing rhythm that resulted in more than an occasional gasp, fleeting palpated pulse, or arterial waveform. Interval from cardiac arrest to ECMO was defined as time from collapse to the point of ECMO setup and administration. ROSC time was defined as the sum of time of ROSC before ECMO.

**Table 1:** **Glasgow-Pittsburgh Cerebral Performance Categories**

Cerebral performance categories*	
**1. Good cerebral performance**	Conscious: Alert, able to work and lead a normal life. May have minor psychological or neurological deficits (mild dysphasia, nonincapacitating hemiparesis, or minor cranial nerve abnormalities).
**2.Moderate cerebral disability**	Conscious. Sufficient cerebral function for part-time work in sheltered environment or independent activities of daily life (dressing, traveling by public transportation, preparing food). May have hemiplegia, seizures, ataxia, dysarthria, dysphasia, or permanent memory or mental changes.
**3.Severe cerebral disability**	Conscious. Dependent on others for daily support because of impaired brain function (in an institution or at home with exceptional family effort). At least limited cognition. Includes a wide range of cerebral abnormalities from ambulatory with severe memory disturbance or dementia precluding independent existence, to paralytic and able to communicate only with eyes (locked-in syndrome).
**4. Coma, vegetative state**	Not conscious. Unaware of surroundings, no cognition. No verbal or psychological interactions with environment.
**5. Death**	Certified brain dead or dead by traditional criteria.

The ECMO team consisted of cardiovascular surgeons, cardiologists, intensivists, special nurses, and perfusionists. Percutaneous cannulation was established in the femoral vein and artery by Seldinger’s technique. When percutaneous puncturing the femoral artery was difficult, femoral cutdown procedures were performed. Our ECMO flow was described in a previous report [6]. The institutional rapid response team called the oncall ECMO team leader when CPR was performed for more than 10 minutes or for unstable vital signs or recurrent cardiac arrest. The ECMO team leader and CPR leader assessed the patient and made a decision about ECPR. ECPR was performed when cardiopulmonary arrest was persistent despite conventional CPR over 10 minutes, witness arrest was confirmed, and the event that caused arrest was thought to be reversible. Generally, ECPR was performed when probability of reversible cardiac etiology was high or cardiac arrest had a respiratory cause. ECPR was deferred in cases of short life expectancy (<6 months), uncontrolled terminal malignancy, unwitnessed collapse, limited activity, unprotected airway, or more than 60 minutes of CPR time at initial contact. Age alone was not a contraindication for ECPR.

The Capiox Emergency Bypass System (Terumo, Tokyo, Japan) or Prolonged Life Support System (Maquet Cardiopulmonary AG, Hirrlingen, Germany) were used in all cases. Priming used a crystalloid solution such as normal saline or balanced solution. No patients in had blood-primed ECMO. Anticoagulation was accomplished by unfractionated heparin bolus injection followed by continuous intravenous heparin infusion to maintain an activated clotting time between 150 and 200 seconds.

### Statistical analysis

Continuous variables were expressed as medians and interquartile ranges (IQRs). Chi-square or Fisher’s exact test were used as appropriate for categorical data and the Mann–Whitney *U* test was used for continuous data. A multivariate logistic regression model was used to identify predictors of neurological outcomes. All variables associated with neurological outcomes were analyzed by univariate analysis. Factors with *P* < 0.2 that were considered clinically relevant were included in multivariate analysis. SPSS 20.0 software (SPSS Inc., Chicago, IL, USA) was used for statistical analysis. A *P*-value of less than 0.05 was considered statistically significant.

## Results

### Patient characteristics

The median patient age was 58 (range 45–66) years, and 80 patients (70%) were men. Interval from cardiac arrest to ECMO was 34 (range 20–53) minutes. Cardiac etiology was verified in 74 (64%) patients. Acute myocardial infarction was the cause of cardiac arrest in 40 patients (35%) and 38 (33%) had a history of ischemic heart disease. Respiratory etiology was verified in 5 patients (4%). Most patients (83%) had cardiac arrest in the hospital and the few (17%) who had cardiac arrest in a non-hospital setting had ECMO. Patient characteristics are in Table 2.

**Table 2:** **Baseline characteristics of successful ECPR group and univariate analysis for factors associated with good and poor CPC groups**

	Overall	Good CPC group	Poor CPC group	*P-value*
Age (years)	58.0 (45.0-66.0)	58.0 (44.0-66.0)	58.5 (44.5-69.5)	0.32
Gender: Male (%)	80 (70)	47 (69)	33 (70)	0.90
Height (cm)	164.5 (157.0-170.0)	168.0 (157.0-173.0)	163.0 (155.5-169.5)	0.59
Weight (kg)	62.0 (55.1-69.7)	62.0 (54.9-71.0)	63.2 (55.9-69.6)	0.82
Comorbidities				
DM (%)	51 (44)	30 (44)	21 (45)	0.95
HTN (%)	45 (39)	27 (40)	18 (38)	0.88
Smoking (%)	49 (43)	29 (43)	20 (43)	0.99
CKD (%)	8 (7)	6 (9)	2 (4)	0.47
PAOD (%)	4 (4)	2 (3)	2 (4)	0.71
Dyslipidemia (%)	11 (10)	8 (12)	3 (6)	0.52
Acute coronary syndrome	44 (38)	30 (44)	14(30)	0.12
STEMI (%)	22 (19)	17 (25)	5 (11)	
NSTEMI (%)	18 (16)	9 (13)	9 (19)	
Unstable angina (%)	4 (4)	4 (6)	0 (0)	
Location of arrest				
In hospital (%)	96 (83)	61 (90)	35 (75)	
Out of hospital (%)	19 (17)	7 (10)	12 (26)	0.03
Location of ECMO insertion				
Intensive care unit	48 (42)	29 (43)	19 (40)	0.81
Catheterization laboratory room	44 (38)	27 (40)	17 (36)	0.70
Emergency room	14 (12)	7 (10)	7 (15)	0.46
Operating room	7 (6)	5 (7)	2 (4)	0.70
General ward	2 (2)	0 (0)	2 (4)	0.82
Bystander CPR (%)	9 (8)	6 (9)	3 (6)	0.74
Therapeutic hypothermia (%)	9 (8)	5 (7)	4 (9)	0.82
Shockable rhythm (VF/VT) (%)	48 (42)	33 (49)	15 (32)	0.08
Laboratory data				
Hemoglobin before ECMO (g/dL)	11.9 (10.3-14.3)	11.9 (10.2-14.8)	10.7 (9.2-12.3)	0.07
Hemoglobin after ECMO (g/dL)	10.4 (9.2-12.9)	10.0 (8.6-12.4)	9.7 (8.6-12.2)	0.13
T-bil (mg/dL)	0.9 (0.6-1.5)	0.8 (0.6-1.5)	0.9 (0.7-1.2)	0.60
BUN (mg/dL)	17.4 (12.5-24.6)	16.9 (13.8-28.0)	17.0 (11.4-22.1)	0.74
Cr (mg/dL)	1.1 (0.9-1.5)	1.2 (0.9-1.5)	1.2 (0.9-1.7)	0.65
Lactic acid (mmol/L)	8.5 (4.4-13.2)	8.0 (3.9-11.7)	13.6 (6.7-16.5)	0.002
CPR duration (min)	23.0 (15.0-41.0)	19.0 (15.0-28.0)	29.0 (21.0-51.0)	0.01
ROSC before ECMO insertion (%)	50 (44)	31 (46)	19 (40)	0.54
ROSC time (min)	0 (0–5.0)	0 (0–11.0)	0 (0–0)	0.54
Interval from cardiac arrest to ECMO (min)	34.0 (20.0-53.0)	24.5 (18.0-36.0)	40.0 (25.0-60.0)	0.01

ECPR, extracorporeal cardiopulmonary resuscitation; CPC, cerebral performance category; DM, diabetes mellitus; HTN, hypertension; CKD, chronic kidney disease; PAOD, peripheral arterial occlusive disease; STEMI, ST elevation myocardial infarction; NSTEMI, non-ST elevation myocardial infarction; ECMO, extracorporeal membrane oxygenation; CPR, cardiopulmonary resuscitation; VF, ventricular fibrillation; VT, ventricular tachycardia; Hb, hemoglobin; T-bil, total bilirubin; BUN, blood urea nitrogen; Cr, creatinine; ROSC, return of spontaneous circulation.

### ECPR and neurological outcomes

**Figure 2:**
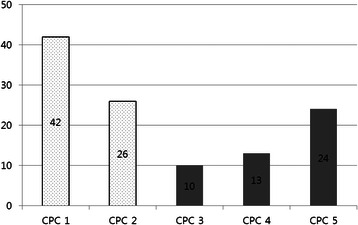
**Cerebral performance category score distribution in good and poor neurological outcomes.**

Of 115 patients, 68 (59%) had good neurological outcomes but 47 (41%) did not (Figure 2). Therapeutic hypothermia was performed in 10 patients (5%). Mean duration of ECMO support was 47.5 (range 18.5–101) hours. Total length of stay in intensive care unit (ICU) was 11 (range 7–22.5) days and 24 patients died from brain death. Univariate analysis showed no differences between the good and poor neurological outcome groups for age, comorbidities, bystander CPR, therapeutic hypothermia, total bilirubin, creatinine, 24-hour lactic acid clearance, ROSC before ECMO, or ROSC time (Table 2).

**Figure 3:**
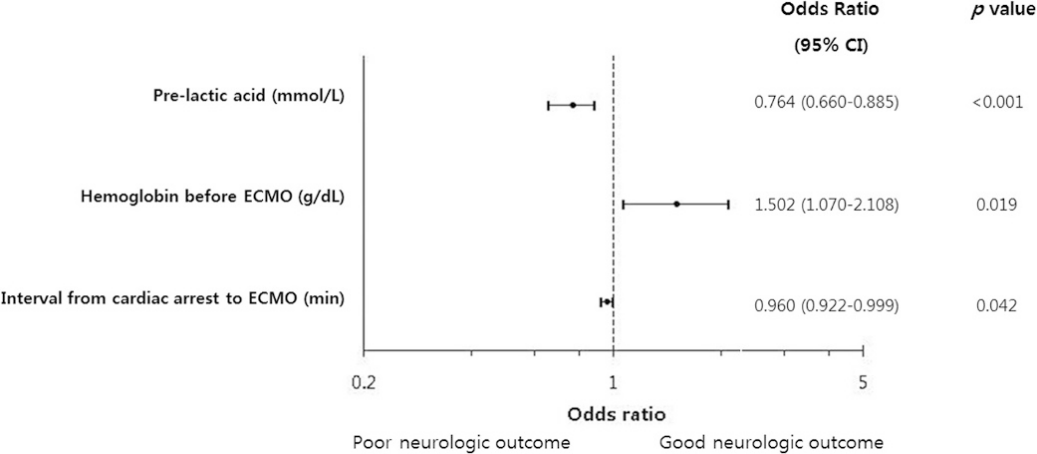
**Independent predictors of good neurological outcomes.** Pre-lactic acid, lactic acid level before ECMO; ECMO, extracorporeal membrane oxygenation; CI, confidence interval.

Multivariate analysis revealed neurological outcomes were affected by hemoglobin level, serum lactic acid before ECMO insertion, and interval from cardiac arrest to ECMO (Figure 3). However, age, gender, cardiac arrest out of the hospital, hemoglobin level after ECMO, acute coronary syndrome, initial shockable rhythm, and CPR duration were not independent predictors of neurological outcomes (Table 3).

**Table 3:** **Multivariate analysis for factors associated with good neurological outcomes**

	OR	(95 % CI)	*P*-value
Age (years)	0.965	(0.931-1.001)	0.06
Gender Male (%)	0.576	(0.139-2.358)	0.45
Pre-lactic acid (mmol/L)*	0.764	(0.660-0.885)	<0.001
Out of hospital CPR (%)	0.141	(0.017-1.187)	0.07
Hemoglobin before ECMO (g/dL)*	1.502	(1.070-2.108)	0.02
Hemoglobin after ECMO (g/dL)	0.932	(0.720-1.207)	0.59
Acute coronary syndrome (%)	3.387	(0.677-16.938)	0.14
Shockable rhythm (VF/VT) (%)	3.679	(0.881-15.360)	0.07
CPR duration (min)	1.003	(0.967-1.041)	0.86
Interval from cardiac arrest to ECMO (min)*	0.960	(0.922-0.999)	0.04

*Variables with *P* < 0.05; OR, odds ratio; CI, confidence interval; CPR, cardiopulmonary resuscitation; ECMO, extracorporeal membrane oxygenation; VF, ventricular fibrillation; VT, ventricular tachycardia.

## Discussion

We excluded 90 patients whose neurological outcomes could not be assessed or who died from unknown causes or causes other than brain death. Neurological status could not be assessed for a few patients because of poor medical records. Death from unknown causes or causes other than brain death was not possible to define as meaningful CPC 5, because this study concerns ECPR and neurological outcomes instead of morbidity and mortality. The study included 115 patients.

Chest compression results in 25–30% normal cardiac output when performed under optimal conditions [4]. Prolonged CPR duration is related to cerebral damage and low chance of ROSC [9]. However, ECMO can rapidly normalize both blood flow and oxygenation. If successful, ECMO can effectively protect major organs and often reverse the underlying cause of cardiac arrest [4,5,10,11].

However, ECPR has two major limitations. One is the need for anticoagulation. ECPR should not be performed in patients with contraindications of anticoagulation such as intracranial bleeding. The other is limited availability and time for preparation and insertion. ECPR devices must be kept ready for use. ECMO team leaders should be familiar with both surgical and catheter techniques. ECMO in patients undergoing CPR can take longer and carry higher risks of procedural failure and complications. A long time between cardiac arrest and ECMO “pump on” may be tolerated by many major organs except the brain, which is the organ most sensitive to hypoxia. Thus, poor neurological outcomes with preserved function of vital organs other than the brain can result from ECPR. Liberal use of ECMO as a CPR method risks increasing the number of patients with severe neurological deficits who require large amounts of familial and social resources. Although several factors, including initial rhythm, resuscitation duration, underlying causes, and initial resuscitative efforts are associated with both survival and neurological outcomes after ECPR [11], we focused on predictors of favorable neurological outcomes after successful ECPR.

Hemoglobin level before ECMO insertion was an important factor for neurological outcomes after successful ECPR. We found no studies that investigated the relationship between hemoglobin level and neurological outcomes after ECPR. Initial hemoglobin level is reported as important for neurological outcomes after conventional CPR [12]. The SOS-KANTO study showed higher hemoglobin levels at hospital arrival are associated with favorable short-term neurological outcomes among postcardiac-arrest patients [12]. Animal studies also showed that the penumbral brain is more vulnerable than the normal brain, with oxygen delivery and cerebral metabolic rate progressively declining as hemoglobin concentration decreases [13,14]. These mechanisms appear to be physiological neuroprotective mechanisms against ischemia caused by anemia. For ECPR patients, most had prolonged cardiac arrest with bolus infusion of crystalloid and colloid fluids. Because the crystalloid solution for ECMO priming dilutes the blood, hemoglobin levels can decrease acutely. Low hemoglobin levels before ECMO insertion might be associated with poor neurological outcomes. Efforts to correct anemia before or during ECMO administration could enhance oxygen delivery and be neuroprotective. Although high hemoglobin levels were associated with good neurological outcome after ECPR in our study, no investigations have examined if higher hemoglobin levels from transfusions are neuroprotective. One hypothesis states that regular blood transfusion may prevent silent ischemia and cognitive impairment in patients with septic shock [15]. A controlled trial by DeBaun et al. showed that regular blood-transfusion therapy significantly reduced the incidence of recurrence of silent and overt cerebral infarction in specific populations (e.g., patients with sickle cell anemia, who require dialysis, or have β-thalassemia) [16]. Recurrence of silent and overt cerebral infarction is associated with cognitive impairment. Transfusions have side effects and tolerating lower transfusion triggers results in comparable or even better outcomes, as shown multiple studies in other settings. Thus, the association of good neurological outcomes with higher hemoglobin levels induced by transfusion requires further investigation.

Lactic acid is a useful marker of tissue hypoxia. Levels of lactic acid and its clearance from the blood are associated with mortality and neurological outcomes after CPR [17]. Kliegel et al. showed that persistent hyperlactemia for 48 hours predicts mortality and poor neurological outcomes after conventional CPR [18]. Lactic acid level and interval from cardiac arrest to ECMO are significantly correlated [19]. Sawamoto et al. reported a significant difference in serum lactic acid level between patients with good and poor neurological outcomes who had primary hypothermic cardiac arrest resuscitated with ECPR [20]. This study showed neurological outcomes are associated with serum lactic acid levels before ECMO insertion. Several studies showed CPR duration is associated with survival rate and neurological outcomes [9,21].

Our analysis showed interval from cardiac arrest to ECMO was prognostic, but CPR duration was not. CPR duration did not include ROSC time. Since ROSC does not necessarily mean stable vital signs with good oxygen delivery, patients can still be hypotensive and hypoxic from myocardium damage and pulmonary problems. Therefore, in ECPR, brain perfusion can be affected by interval from cardiac arrest to ECMO rather than CPR duration. Thus, using ECMO without hesitation might save brain function when a patient is still unstable after ROSC.

This study had several limitations. CPC scale was retrospectively determined based on medical records. We excluded patients whose neurological status could not be assessed because of deterioration followed by death. However, we included patients who had a diagnosis of brain death. Therapeutic hypothermia may be associated with neurological outcomes after cardiac arrest [22]. Therapeutic hypothermia was performed on a limited number of patients in our study, for three reasons. First, hypothermia can cause coagulopathy, so therapeutic hypothermia was not tried if patients had substantial bleeding after ECMO insertion. Second, controlled surface cooling devices were not available before 2009 in our hospital. From 2010, we have selectively used therapeutic hypothermia in ECPR patients. Third, most ECPR patients had low body temperature caused by extracorporeal circulation and external volume infusion. Therefore, ECMO itself could have some degree of neuroprotective effect through hypothermia. Although a few studies investigated ECPR and neurological outcomes, most included all ECPR patients [4,10,11] and did not suggest prognostic factors for favorable neurological outcomes. Those studies also might have included patients who died during ECMO or soon after ECMO weaning. Lacking a definition for ECMO procedural success or salvage, we excluded patients who did not survive for more than 24 hours after ECMO initiation.

## Conclusions

In conclusion, low hemoglobin, high serum lactic acid levels, prolonged interval from cardiac arrest to ECMO were independent predictors for poor neurological outcomes after successful ECPR. Early ECMO institution, low blood transfusion threshold, and a sophisticated EMCO program that enables accurate ECPR decisions and fast ECMO procedures may improve patient neurological outcomes for patients who survive cardiac arrest.

## Abbreviations

ECMO: Extracorporeal membrane oxygenation
CPR: Cardiopulmonary resuscitation
ECPR: Extracorporeal cardiopulmonary resuscitation
ROSC: Recovery of spontaneous circulation
CPC: Cerebral performance categories scale
IQR: Interquartile range
ICU: intensive care unit
